# Auricular Acupressure in Patients with Hypertension and Insomnia: A Systematic Review and Meta-Analysis

**DOI:** 10.1155/2020/7279486

**Published:** 2020-06-17

**Authors:** Zhi-Hui Zhao, Yi Zhou, Wei-Hong Li, Zhao-Hui Tang, Ting-Wei Xia, Han-Li

**Affiliations:** ^1^Basic Medical College, Chengdu University of TCM, Chengdu, Sichuan 611137, China; ^2^Hospital of Chengdu University of TCM, Chengdu, Sichuan 610072, China

## Abstract

**Objective:**

The efficacy of auricular acupressure in patients with hypertension and insomnia is controversial. This systematic review aims to explore the effectiveness of auricular acupressure in reducing blood pressure and improving sleep in this population.

**Methods:**

We conducted an extensive database search in Cochrane Central Register of Controlled Trials, PubMed, Ovid LWW, Web of Science, Chinese Biomedical Literature Database, China Knowledge Resource Integrated Database, Wanfang Data, and China Science and Technology Journal Database on randomized controlled trials published from inception to November 2019 that compared auricular acupressure with a control or comparison group on blood pressure control and sleep improvement. Two reviewers independently conducted data screening and extraction. Study quality was evaluated using the *Cochrane Handbook for Systematic Reviews of Interventions*. Meta-analyses were performed on blood pressure, Pittsburgh Sleep Quality Index (PSQI), the efficacy rate of diagnostic and therapeutic criteria for traditional Chinese medicine syndromes (DTCTCMS), and the efficacy rate of guidelines for traditional Chinese medicine (new drug) clinical research (GTCMCR) by Revman 5.3.0.

**Results:**

A total of 18 randomized controlled trials with 1685 patients were identified. Compared with a control or comparison group, pooled meta-analyses showed that auricular acupressure significantly improved systolic blood pressure (MD = −15.05, 95% CI (−18.49, −11.61), *P* < 0.00001), diastolic blood pressure (MD = −8.41, 95% CI (−11.33, −5.48), *P* < 0.00001), PSQI (MD = −2.37, 95% CI (−4.64, −0.10), *P*=0.04), the efficacy rate of DTCTCMS (RR = 1.63, 95% CI (1.16, 2.28), *P*=0.004), and the efficacy rate of GTCMCR (RR = 1.25, 95% CI (1.12, 1.38)).

**Conclusions:**

The results demonstrated a favorable effect of auricular acupressure to reduce blood pressure and improve sleep in patients with hypertension and insomnia. Further studies to better understand the acupoints and intervention times of auricular acupressure are warranted.

## 1. Introduction

The incidence of hypertension is gradually increasing due to changes in living conditions and habits. A significant proportion of hypertensive patients are accompanied by insomnia [1]. Evidence suggests that the shortening of sleep time is closely linked to hypertension in young, middle-aged [2], and elderly patients [3]. Shortened sleep times may affect hypertension in many ways, with effects on the autonomic nervous system [4, 5], inflammatory factors [6, 7], and the quality of life of patients. Vgontzas et al. believed that sustained insomnia may even become an independent risk factor for hypertension [8]. Antihypertensives combined with sleeping drugs comprise the current mainstream treatment [9], but sleeping pills are often not accepted by patients because they may develop dependency.

As an essential branch of traditional Chinese medicine (TCM) treatment, auricular acupressure has been widely used in various diseases, such as obesity [10], anxiety [11], pain [12], and insomnia [13]. In this context, the efficacy of auricular acupressure on hypertension and insomnia has gradually been recognized, and many researchers have also explored this linkage. However, their results are questionable due to the small sample sizes and different measurement standards in each study. In this study, the therapeutic effects of auricular acupressure on hypertension and insomnia were systematically evaluated, and a meta-analysis was conducted to provide a reference for the treatment of these conditions.

## 2. Method

The method used in this systematic review has been previously registered in PROSPERO (CRD42020153992), which is available from https://www.crd.york.ac.uk/prospero/.

### 2.1. Data Sources and Search Strategies

The following databases were searched using a computer: Cochrane Central Register of Controlled Trials (CENTRAL), PubMed, Ovid LWW, Web of Science, Chinese Biomedical Literature Database (CBM), China Knowledge Resource Integrated Database (CNKI), Wanfang Data, and China Science and Technology Journal Database (VIP). The search strategy terms included auricular acupressure, high blood pressure, insomnia, sleep disorders, convulsions, and dizziness. The retrieval time was from the establishment of the database to November 2019, and the references to the included articles were also retroactively searched. An illustrative PubMed search strategy is as follows:

Hypertension OR Blood Pressure, High OR Blood Pressures, High OR High Blood Pressure OR High Blood Pressures
Disorders of Initiating and Maintaining Sleep OR DIMS (Disorders of Initiating and Maintaining Sleep) OR Early Awakening OR Awakening, Early OR Nonorganic Insomnia OR Insomnia, Nonorganic OR Primary Insomnia OR Insomnia, Primary OR Transient Insomnia OR Insomnia, Transient OR Rebound Insomnia OR Insomnia, Rebound OR Secondary Insomnia OR Insomnia, Secondary OR Sleep Initiation Dysfunction OR Dysfunction, Sleep Initiation OR Dysfunctions, Sleep Initiation OR Sleep Initiation Dysfunctions OR Sleeplessness OR Insomnia Disorder OR Insomnia Disorders OR Insomnia OR Insomnias OR Chronic Insomnia OR Insomnia, Chronic OR Psychophysiological Insomnia OR Insomnia, Psychophysiological
Auricular seed pressing OR Auricular point pressing OR Auricular pressing OR Auricular acupoint pressing OR Auricular acupressure OR Auricular seed acupressure OR Auricular point acupressure
(1) and (2) and (3)

### 2.2. Criteria for considering Studies for This Review

Studies should meet the following inclusion criteria (PICO format). (1) Participants: this refers to patients with a clear diagnosis of hypertension and insomnia. The diagnostic criteria for hypertension are as referred to in China's Guidelines for Prevention and Treatment of Hypertension (2018 Revision) [14]. And the following criteria are referenced in the diagnosis of insomnia: American diagnostic and statistical manual of mental disorders, fifth edition (DSM-V) [15], international classification of diseases-10 (ICD-10) [16], international classification of sleep disorders (ICSD) [17], classification and diagnostic criteria for Chinese mental disorders (CCDM) [18], the diagnostic and therapeutic criteria for traditional Chinese medicine syndromes (DTCTCMS) [19], guidelines for traditional Chinese medicine (new drug) clinical research (GTCMCR) [20], and other commonly used diagnostic criteria. The age, gender, race, source of case, time of illness, etc. of subjects were not restricted. (2) Interventions: the experimental group was given auricular acupressure, and the manipulation and specific acupoints were not restricted. (3) Control: this refers to any type of control group, including conventional Western medicine, routine nursing, or blank control. (4) Outcomes: at least one of the following scales was required to be included in the evaluation of sleep quality: Pittsburgh Sleep Quality Index (PSQI) [21], the efficiency of the diagnostic and therapeutic criteria for TCM syndromes [19], the efficiency of guidelines for TCM (new drug) clinical research [20], sleep status self-assessment scale [22], or other inferable data mentioning insomnia and auricular acupressure for carrying out meta-analysis; and systolic and diastolic blood pressure were used to evaluate blood pressure. (5) Study type: the type of study was randomized controlled trials.

Exclusion criteria were the following: (1) duplicate literature; (2) incomplete literature information; (3) differences in baseline between groups; (4) no exact efficacy evaluation index.

### 2.3. Literature Screening and Data Extraction

First, two investigators reviewed the titles and abstracts independently according to the preset inclusion and exclusion criteria, and then they read the full texts after excluding apparently unrelated literature. The final included literature was identified after further screening, and after that, two investigators extracted the data while blinded to each other's review. Finally, the results were cross-checked. The differences were resolved by consensus with a third investigator.

The data extraction included the following aspects: (1) general information: first author, publication year, literature topics, etc.; (2) research characteristics: baseline comparability, sample size, sex ratio, country, intervention measures, treatment course, and follow-up; (3) outcome indicators; and (4) relevant factors for evaluating the risk of bias.

### 2.4. Assessment of Risk of Bias in Included Studies

Two reviewers independently assessed the risk of bias in accordance with suggested categories listed in the *Cochrane Handbook for Systematic Reviews of Interventions* [23] and then conducted cross-checking. The differences were resolved by consensus with a third investigator. RevMan5.3.0, provided by the Cochrane Collaboration, was used to created plots demonstrating the risks of bias.

### 2.5. Statistical Analysis

Rewman 5.3.0 was used for statistical analysis. The mean difference (MD) and 95% confidence interval (95% CI) were used when the outcomes were continuous variables, while the risk ratio (RR) and 95% CI were used when the outcomes were two categorical variables. The chi-square test and *I*^2^ statistic were used to check the heterogeneity of the results. The fixed-effects model was used when the statistical heterogeneity was small (*P* ≥ 0.1, *I*^2^ < 50%); otherwise, subgroup analyses according to total sample size, intervention time, control type were performed, as well as sensitivity analyses if necessary. Publication bias was estimated with a funnel plot.

## 3. Results

### 3.1. Literature Search Results

**Figure 1:**
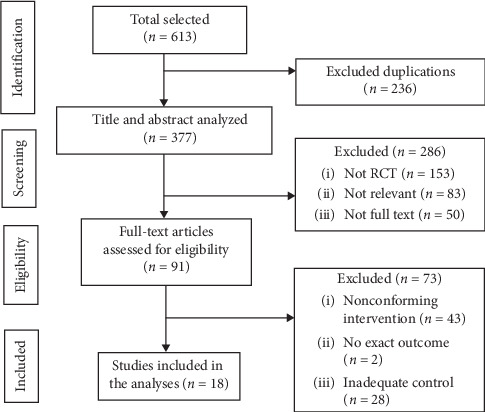
Flow chart of selection process.

613 original studies were collected by database searching, and 236 duplicate studies were excluded. After screening the titles and abstracts of the remaining literature, 286 articles were excluded, including case reports, reviews, and irrelevant publications. Then the full texts were read, and another 73 studies were excluded due to their nonconforming interventions, inadequate control groups, and inaccurate evaluation indicators. At last, 18 studies were included [24–41]. The specific screening process and results are displayed in Figure 1.

### 3.2. Characteristics of the Included Literature

A total of 18 studies were included with a total of 1685 patients. Their basic characteristics are shown in Table 1. In these 18 studies, the experimental group underwent auricular acupressure, among which 13 studies [24, 27, 29–32, 34, 35, 37, 39, 41] had an intervention time of ≤15 days, and 5 studies [25, 26, 33, 36, 38] had an intervention time of >15 days. The intervention measures of the control groups were as follows: routine nursing was used in 8 studies [28, 30, 31, 33, 34, 37–39], conventional Western medicine was used in 8 studies [24–27, 29, 32, 36, 41], and no intervention was used in 2 studies [35, 40]. The outcomes included were as follows: PSQI was used in 10 studies [26–28, 30–34, 36, 38], the diagnostic and therapeutic criteria for TCM syndromes were used in 3 studies [35, 40, 41], the guidelines for TCM (new drug) clinical research were used in 3 trials [24, 32, 37], Sleep Status Self-Assessment Scale was used in 2 trials [29, 39], and an improved Sleep Status Self-Assessment Scale was used in 1 study [25]. Blood pressure was measured in 10 studies [25, 26, 28, 30, 33–36, 38, 40].

### 3.3. Methodological Quality of the Included Studies

**Figure 2:**
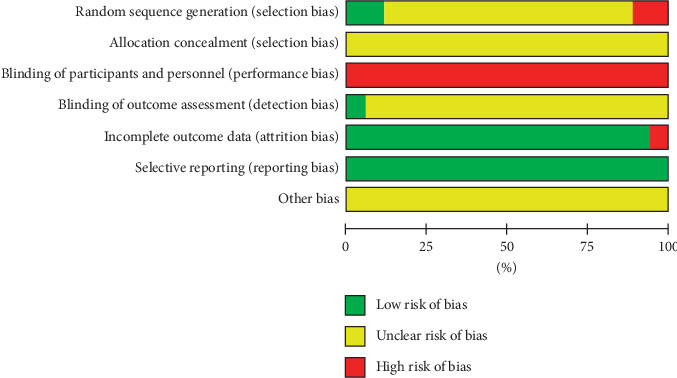
Risk of bias graph.

**Figure 3:**
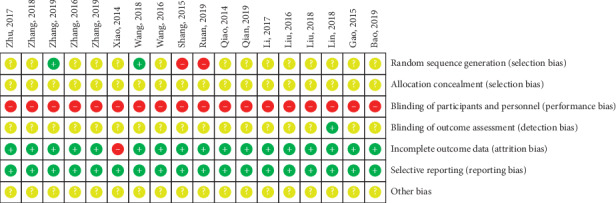
Risk of bias summary.

The risk of bias assessment of all included studies is shown in Figures 2 and 3. Two studies that reported the method of randomization in terms of random number table were accorded a low risk of bias in random sequence generation [24, 30]. Two studies constituted a high risk of bias on account of the alternate allocation method (odd or even hospitalization date) used in these trials [26, 38]. The remaining 14 studies were classified as having an unclear risk of bias because insufficient information was provided [25, 27–29, 31–37, 39–41]. None of 18 studies described the process of allocation concealment in sufficient detail and were judged as having an unclear risk of bias. Due to the obvious difference-whether to use auricular acupressure or not-between the auricular acupressure group and the control group, a blind method could not be used in the participants or administrators in any of the 18 studies [24–41]. One study [32] was shown to blind its outcome assessment and was graded as low risk of bias. One study [35] that had a high drop-out rate in the control group was judged to have a high risk of bias for incomplete outcome data, and the remaining 17 studies [24–34, 36–41] were classified as having a low risk of bias because all the pre-set outcomes were reported. All studies reported all outcomes listed in their methods section and were graded as low risk of bias for selective reporting. The data necessary for judging the risk of other biases were insufficient in 18 studies [24–41].

## 4. Outcomes

### 4.1. Outcomes Related to Blood Pressure

#### 4.1.1. Systolic Blood Pressure

**Figure 4:**
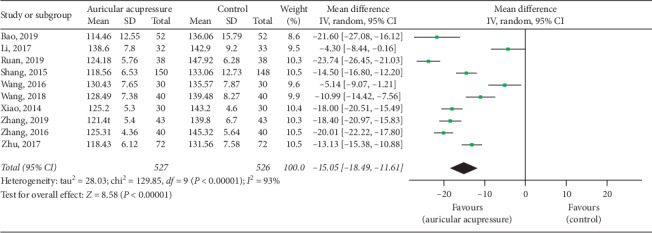
Forest plot of the comparison between auricular acupressure and the control group for the outcome systolic blood pressure.

Systolic blood pressure was examined in 10 articles [25, 26, 28, 30, 33–36, 38, 40]. The pooled results showed that auricular acupressure significantly reduced systolic blood pressure (MD = −15.05, 95% CI: [−18.49, −11.61], *P* < 0.00001) (Figure 4). Given the high heterogeneity (*I*^2^ = 93%, *P* < 0.00001), sensitivity analyses were performed to explore potential sources of heterogeneity, and the results did not change substantively. The heterogeneity ranged from 92% to 94%. Then, subgroup analyses were conducted based on total sample size (≤80 cases and >80 cases), comparison method (auricular acupressure plus antihypertensive drugs *vs.* antihypertensive drugs, and auricular acupressure *vs.* antihypertensive drugs), and intervention time (≤15 days and >15 days). The results showed a significant difference of heterogeneity in subgroups of comparison method, indicating that the difference of the comparison method was partly the reason why there was severe heterogeneity in the overall analysis (Table 2).

#### 4.1.2. Diastolic Blood Pressure

**Figure 5:**
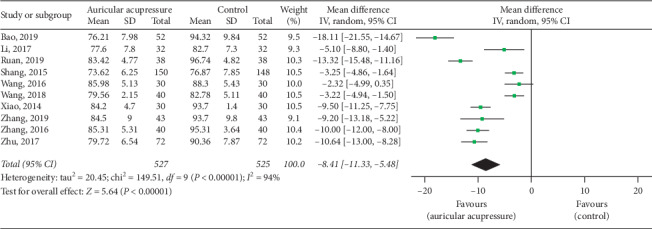
Forest plot of the comparison between auricular acupressure and the control group for the outcome diastolic blood pressure.

Ten studies [25, 26, 28, 30, 33–36, 38, 40] reported diastolic blood pressure levels. The pooled result also showed a significant difference between auricular acupressure and the control group in reducing diastolic blood pressure (MD = −8.41, 95% CI: [−11.33, −5.48], *P* < 0.00001), with high heterogeneity (*I*^2^ = 94%, *P* < 0.00001) (Figure 5). Then, we carried out sensitivity analyses, and the heterogeneity remained high (ranged from 93% to 95%). Thus, subgroup analyses based on total sample size (≤80 cases and >80 cases), comparison method (auricular acupressure plus antihypertensive drugs *vs.* antihypertensive drugs, and auricular acupressure *vs.* antihypertensive drugs), and intervention time (≤15 days and >15 days) were conducted. A significant difference in heterogeneity was found in the subgroup of comparison method, indicating that the difference of comparison method was partially the reason for severe heterogeneity. The detailed results are shown in Table 3.

### 4.2. Relevant Measures Related to Insomnia

#### 4.2.1. PSQI

**Figure 6:**
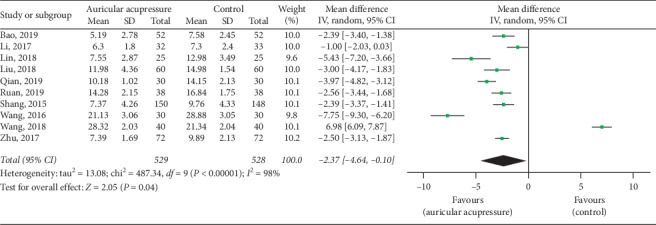
Forest plot of the comparison between the auricular acupressure and the control group for the outcome PSQI.

A total of 10 articles with 1057 patients [26–28, 30–34, 36, 38] used PSQI scores as an outcome. The pooled result suggested that the auricular acupressure was superior to the control group in the improvement of the PSQI (MD = −2.37, 95% CI: [−4.64, −0.10], *P*=0.04), with high heterogeneity (*I*^2^ = 98%, *P* < 0.00001) (Figure 6). So, we carried out sensitivity analyses and subgroup analyses to investigate the potential sources of heterogeneity. The heterogeneity did not change after the studies were removed one by one, which means that the heterogeneity result was stable. We also carried out subgroup analyses according to the total sample size(≤80 cases and >80 cases), intervention time(≤15 days and >15 days), and comparison method(auricular acupressure plus routine nursing *vs.* routine nursing, and auricular acupressure plus hypnotics *vs.* hypnotics). Subgroup analyses showed significant results between subgroups of total sample size ≤80 cases and >80 cases, which may be partly the reason why there was severe heterogeneity in the overall analysis (Table 4).

In addition, the pooled results of subgroup analyses showed significant improvement of PSQI in auricular acupressure compared to the control group for intervention time ≤15 days (MD = −4.16, 95% CI: [−6.20, −2.13], *P* < 0.0001) rather than intervention time >15 days (MD = −0.12, 95% CI: [−4.56, 4.32], *P*=0.96), as well as the comparison method of auricular acupressure plus hypnotics *vs.* hypnotics (MD = −4.67, 95% CI: [−7.09, −2.25], *P*=0.00002) rather than the method of auricular acupressure plus routine nursing *vs.* routine nursing (MD = −0.71, 95% CI: [−3.86, 2.43], *P*=0.66). However, the heterogeneity of these subgroups was still very high, which may be caused by the low methodological quality of the included studies.

#### 4.2.2. Efficiency Rate of the Diagnostic and Therapeutic Criteria for TCM Syndromes

**Figure 7:**
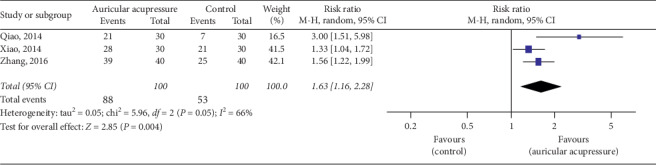
Forest plot of the comparison between auricular acupressure and the control group for the efficiency rate of the diagnostic and therapeutic criteria for TCM syndromes.

The efficiency rate of the diagnostic and therapeutic criteria for TCM syndromes was used in 3 studies [35, 40, 41]. These criteria, promulgated by the State Administration of Traditional Chinese Medicine of China, divides the improvement of insomnia into three levels, that is, cure: sleep returned to normal or sleep time reached 6 hours; improvement: insomnia was significantly improved or sleep time increased by more than 3 hours; ineffectiveness: insomnia did not improve. Effective cases are a summary of cured and improved cases. A random effect model was used for meta-analysis due to high heterogeneity among these studies (*I*^2^ = 66%, *P*=0.05). The results showed that participants who received auricular acupressure had more sleep improvement compared to the control group (RR = 1.63, 95% CI: [1.16, 2.28], *P* < 0.0001) (Figure 7). Then, we carried out sensitivity analyses and found that the heterogeneity changed significantly (*I*^2^ = 66%, *P*=0.50) after removing one set of data [41]. It seemed that this study, showing that auricular acupressure had more significant effects than other studies, may be the potential source of heterogeneity. However, when we looked up the study again, we failed to find differences in methodology and other aspects.

#### 4.2.3. Efficiency Rate of the Guidelines for TCM (New Drug) Clinical Research

**Figure 8:**
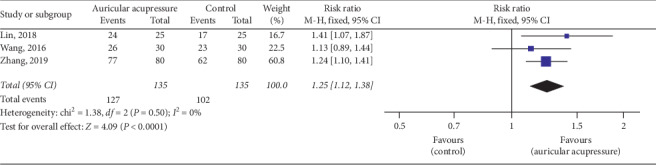
Forest plot of the comparison between auricular acupressure and the control group for the efficiency rate of the guidelines for TCM (new drug) clinical research.

The guidelines for TCM (new drug) clinical research were used in 3 studies [24, 32, 37]. These guidelines, issued by the Chinese Ministry of Health, divides the improvement of insomnia into four levels, that is, cure: noticeable improvement in sleep quality or effective sleep time over 6 hours; significant effectiveness: moderate improvement in sleep quality or effective sleep time up to 3–6 hours; effectiveness: insignificant improvement in sleep quality or sleep time less than 3 hours; ineffectiveness: no improvement in sleep quality or the effective sleep time is not extended. A fixed effects model was used for meta-analysis due to low levels of heterogeneity among these studies (*I*^2^ = 0%, *P* = 0.50). The combined result was statistically significant (RR = 1.25, 95% CI: [1.12, 1.38], *P* < 0.0001) compared to the control group, showing favorable effects of auricular acupressure in sleep improvement (Figure 8).

#### 4.2.4. Sleep Status Self-Assessment Scale

Sleep Status Self-Assessment Scale was used in 2 studies [29, 39], while a self-made Sleep Quality Self-Assessment Scale was used in 1 study [25]. The meta-analysis could not be carried out due to the inconsistency of the data and evaluation standards. However, all 3 studies suggested that the experimental groups were more effective than the control groups in improving sleep quality (*P* < 0.05 for all 3 groups).

### 4.3. Publication Bias

**Figure 9:**
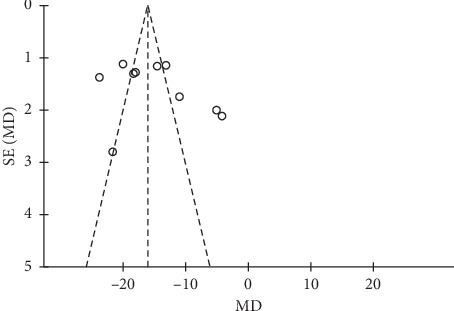
Funnel plot of trials comparing systolic blood pressure.

The funnel plot was drawn based on studies that included the outcome of systolic blood pressure. Six studies [26, 30, 33–36] were significantly asymmetrical, suggesting that publication bias might exist (Figure 9). However, when we looked up these 6 studies again, we did not find differences in methodology and other aspects.

### 4.4. Safety Monitoring

A description of serious adverse events was not found in the included studies.

## 5. Discussion

A total of 18 articles were included in this systematic review. The key finding from this study showed that auricular acupressure may have positive effects on the treatment of patients with hypertension and insomnia, and it may be an augmentation approach to control blood pressure and improve insomnia.

### 5.1. Main Results and Analysis

The incidence of hypertension with insomnia is increasing gradually [42]. In China, due to the resistance to sleeping pills and the desire to take Western medicine as little as possible, many patients seek help from TCM, including pharmaceutical and nonpharmacological treatments. Therefore, auricular acupressure is favored because of its simple operation, and patients can press the acupuncture points at any time when they are free, without the aid of a doctor [43]. In China, auricular acupressure, a crucial nonpharmacological treatment method, is often applied in combination with medications to treat individuals with hypertension and insomnia. Some scholars believe that the regulation of auricular acupressure for hypertension and insomnia is affected by the vagus nerve, which regulates neuroendocrine self-balancing [44]. Fallgater recorded the vagal somatosensory-evoked potential from stimulating the ear armor and proposed that this potential originated from the dorsal nucleus of the brainstem nerve. The dorsal nucleus of the brainstem nerve was shown to be synaptic with the hypothalamus and amygdala to regulate insomnia [45]. In our study, we also found that auricular acupressure may be effective in the above two aspects: sleep improvement and blood pressure reduction. We believe that auricular acupressure may be a complementary treatment for hypertension and insomnia, which can be promoted to other countries.

The results of this meta-analysis showed that a significant improvement of insomnia could be seen in the intervention time of ≤15 days and the comparison method of auricular acupressure plus hypnotics vs. hypnotics. At present, there is no randomized controlled trial proving this conclusion. However, Liu and Wang [46] have proposed the concept of “meridian fatigue,” which refers to the phenomenon that occurs when a disease cannot be cured with long-term treatment or may even get worse due to the excessive consumption of qi and blood in meridians and the weakening of the functioning of meridians and collaterals. Zhang et al. [47] also believed that inappropriate high-intensity stimulation on acupoints might consume vital qi, thus affecting the disease recovery process. However, the interpretation of these results should be treated with caution in clinical practice. In this meta-analysis, high heterogeneity, a relatively small number of studies, low quality of literature, and nonuniform auricular acupressure procedures may have led to this result. A high-quality, well-designed, large-scale trial is still needed to further validate this conclusion.

### 5.2. Limitations

The overall quality of the included studies was not high, with only 2 studies reporting appropriate randomization methods, and none of the 18 studies mentioned allocation concealment. At the same time, all the participants and administrators cannot be blinded to whether to use auricular acupressure or not, and the data for judging the other biases in all 18 studies were inadequate. In addition, publication bias existed. In the funnel plot, six studies were significantly asymmetrical, where, however, differences in methodology and other aspects cannot be found due to the low quality and inadequate information provided in these studies.

The high heterogeneity of the results cannot be ignored either. We conducted subgroup analyses with respect to intervention time, comparison method, and total sample size. We found that the comparison method and total simple size might partly be the source of heterogeneity. However, they can only explain the heterogeneity to some extent, and there remained substantial unexplained heterogeneity in the pooled results, such as study design and study quality. Although auricular acupressure was the primary intervention method, the specific acupoints and operation methods varied between each study. In addition, the inclusion criteria for each study were slightly different, as some included grade 1 hypertensive patients and some were grade 1 and grade 2 hypertensive patients, while others had unrestricted participation. Therefore, the result that the heterogeneity may come from comparison methods and sample sizes should be interpreted with great caution.

Additionally, the included studies were conducted in Asian countries, so the results lack evidence from Western countries. Chinese and English were the 2 basic search languages, but other languages should be searched to expand the scope as well. Moreover, in terms of sleep improvement, subjective scales were adopted by most of the studies, which were less convincing than the objective indicators. And the evidence on the safety evaluation of auricular point pressing is lacking. All these points need further observation and analysis in future research work.

### 5.3. Practical Implications

According to our study, auricular acupressure may be effective for hypertension and insomnia, but the potential findings should be interpreted carefully on account of low quality and clinical heterogeneity. Nevertheless, the quantitative analysis of the current research is still a constructive reference for future clinical trials. From a clinical point of view, the development of the standard auricular acupressure process, frequency of the operation, acupoint combination, and duration of treatment of auricular acupressure are urgent problems to be solved.

## 6. Conclusion

Based on current evidence, auricular acupressure may be effective in patients with hypertension and insomnia. Auricular acupressure can be promoted as a possible alternative method for blood pressure and sleep management in this population. However, due to the limitations of the literature, large-sample, multicenter, well-designed clinical trials are still required. Different acupoints and intervention times may need to be analyzed to further explore the factors affecting the efficacy of the treatment.

## Figures and Tables

**Table 1:** Characteristics of included studies.

Author, year	Participants	No. (T/C)	Main acupoints	Intervention time (days)	Control type	Outcome measures
Shang, 2015	Middle aged and elderly patients with hypertension and insomnia	148/150	Jiangyagou, Shenmen, heart, endocrine, Jiaogan, and Pizhixia	28	I	①②③
Lin, 2018	Young and middle-aged patients with hypertension and insomnia	25/25	Heart, kidney, and liver	6	II	①⑥
Zhang, 2018	Patients with hypertension and insomnia	36/36	Jiangyagou, Shenmen, heart, brain, kidney, liver, and spleen	18	II	⑤
Zhang, 2019	Patients with hypertension and insomnia	43/43	Jiangyagou, Shenmen, Sanjiao, Jiaogan, and Pizhixia	15	II	②③⑦
Gao, 2015	Patients with hypertension and insomnia	30/30	Heart, kidney, brain, Shenmen, and Jiangyagou	15	I	⑦
Wang, 2018	Patients with grades 1 and 2 hypertension and insomnia	40/40	Liver, kidney, Jiaogan, Shenmen, heart, and Jiangyagou	28	I	①②③
Wang, 2016	Patients with grade 1 hypertension and insomnia	30/30	Pizhixia, Jiaogan, Shenmen, and Jiangyagou	14	II	①②③⑥
Zhu, 2017	Patients with hypertension and insomnia	72/72	Jiaogan, Shenmen, Jiangyagou, heart, liver, and kidney	28	I	①②③
Li, 2017	Patients with grades 1 and 2 hypertension and insomnia	32/33	Jiaogan, Shenmen, Pizhixia, heart, and liver	14	I	①②③
Xiao, 2014	Patients with hypertension and insomnia	25/25	Jiaogan, Shenmen, Pizhixia, heart, and endocrine	15	III	②③④
Liu, 2018	Patients with hypertension and insomnia	60/60	Heart, Jiaogan, Shenmen, and Jiangyagou	10	I	①
Qian, 2019	Elderly patients with hypertension and insomnia	30/30	Spleen, liver, Shenmen, heart	14	II	①
Liu, 2019	Elderly patients with hypertension and insomnia	30/30	Heart, Jiaogan, Shenmen, endocrine, Chuiqian, and minor points	10	I	⑤
Ruan, 2019	Patients with hypertension and insomnia	38/38	Heart, liver, kidney, spleen, Jiangyagou	30	II	①②③
Bao, 2019	Patients with hypertension and insomnia	52/52	Heart, liver, kidney, spleen, and Jiangyagou	Unclear	I	①②③
Zhang, 2016	Patients with hypertension and insomnia	40/40	Heart, liver, kidney, spleen, and Jiangyagou	25	III	②③④
Qiao, 2014	Patients with grade 1 hypertension and insomnia	30/30	Liver, kidney, Pizhixia, Jiangyagou, endocrine, and Shenmen	15	II	⑧④
Zhang, 2019	Patients with hypertension and insomnia	80/80	Jiaogan, Shenmen, Jiangyagou, and heart	15	II	⑥

①: PSQI; ②: SBP; ③: DBP; ④: the diagnostic and therapeutic criteria for TCM syndromes; ⑤: Self-Made Sleep Status Self-Assessment Scale; ⑥: guidelines for TCM (new drug) clinical research; ⑦: Sleep Status Self-Assessment Scale. I: routine nursing; II: conventional Western medicine; III: no intervention.

**Table 2:** Subgroup analyses based on various criteria for systolic blood pressure.

Subgroup	*n*	MD (95% CI)	Heterogeneity (%)	*P* value
Total sample size				
>80	6	−16.33 [−19.45, −13.22]	79	*P* < 0.00001
≤80	4	−13.85 [−19.67, −8.04]	96	*P* < 0.00001

Intervention time				
≤15 days	4	−11.62 [−18.75, −4.50]	95	*P* < 0.00001
>15 days	5	−16.52 [−20.81, −12.22]	93	*P* < 0.00001

Control type				
AP + AHDs vs. AHDs	7	−14.42 [−19.16, −9.68]	94	*P* < 0.00001
AP vs. no intervention	2	−19.10 [−21.06, −17.14]	28	*P*=0.24

AP: auricular acupressure; AHDs: antihypertensive drugs.

**Table 3:** Subgroup analyses based on various criteria for diastolic blood pressure.

Subgroup	*n*	MD (95% CI)	Heterogeneity (%)	*P* value
Total sample size				
≤80	6	−7.31 [−10.82, −3.79]	94	*P* < 0.00001
>80	4	−10.21 [−16.56, −3.85]	96	*P* < 0.00001

Intervention time				
≤15 days	4	−6.54 [−10.37, −2.70]	86	*P* < 0.00001
>15 days	5	−8.04 [−12.14, −3.94]	96	*P* < 0.00001

Control type				
AP + AHDs vs. AHDs	7	−8.08 [−11.82, −4.33]	95	*P* < 0.00001
AP vs. no intervention	2	−9.72 [−11.04, −8.40]	0	*P*=0.71

AP: Auricular acupressure; AHDs: Antihypertensive drugs.

**Table 4:** Subgroup analyses based on various criteria for PSQI.

Subgroup	*n*	MD (95% CI)	Heterogeneity (%)	*P* value
Total sample size				
≤80	4	−2.96 [−2.96, −2.09]	0	*P*=0.85
>80	6	−2.26 [−6.42, −1.89]	99	*P* < 0.00001

Intervention time				
≤15 days	5	−4.16 [−6.20, −2.13]	93	*P* < 0.00001
>15 days	4	−0.12 [−12.14, −4.32]	99	*P* < 0.00001

Control type				
AP + AHDs vs. AHDs	6	−0.71 [−3.86, −2.43]	99	*P* < 0.00001
AP vs. no intervention	3	−4.67 [−7.09, −2.25]	84	*P* < 0.00001

AP: auricular acupressure; RN: routine nursing.

## Data Availability

The data used to support the findings of this study are included within the article.
